# Genomics of *Plasmodium vivax* in Colombia reveals evidence of local bottle-necking and inter-country connectivity in the Americas

**DOI:** 10.1038/s41598-023-46076-1

**Published:** 2023-11-13

**Authors:** Edwin Sutanto, Zuleima Pava, Diego F. Echeverry, Tatiana M. Lopera-Mesa, Lidia Madeline Montenegro, Maria F. Yasnot-Acosta, Ernest Diez Benavente, Richard D. Pearson, Sócrates Herrera, Myriam Arévalo-Herrera, Hidayat Trimarsanto, Angela Rumaseb, Rintis Noviyanti, Dominic P. Kwiatkowski, Ric N. Price, Sarah Auburn

**Affiliations:** 1 Exeins Health Initiative, Jakarta, Indonesia; 2 Menzies School of Health Research and Charles Darwin University, Darwin, Australia; 3 Departamento de Microbiología, Universidad del Valle, Cali, Colombia; 4 International Training and Medical Research Center (CIDEIM), Cali, Colombia; 5 Universidad de Antioquia, Medellín, Colombia; 6 Grupo de Investigaciones Microbiológicas y Biomédicas de Córdoba (GIMBIC), Universidad de Córdoba, Monteria, Colombia; 7 Laboratory of Experimental Cardiology, Department of Cardiology, University Medical Center Utrecht, Utrecht, the Netherlands; 8 Wellcome Sanger Institute, Cambridge, UK; 9 Caucaseco Scientific Research Center, Cali, Colombia; 10 Centro Internacional de Vacunas, Cali, Colombia; 11 Eijkman Institute for Molecular Biology, Jakarta, Indonesia; 12 Mahidol‐Oxford Tropical Medicine Research Unit, Mahidol University, Bangkok, Thailand; 13 Centre for Tropical Medicine and Global Health, Nuffield Department of Medicine, University of Oxford, Oxford, UK

**Keywords:** Malaria, Population genetics, Genomics

## Abstract

Colombia aims to eliminate malaria by 2030 but remains one of the highest burden countries in the Americas. *Plasmodium vivax* contributes half of all malaria cases, with its control challenged by relapsing parasitaemia, drug resistance and cross-border spread. Using 64 Colombian *P. vivax* genomes collected between 2013 and 2017, we explored diversity and selection in two major foci of transmission: Chocó and Córdoba. Open-access data from other countries were used for comparative assessment of drug resistance candidates and to assess cross-border spread. Across Colombia, polyclonal infections were infrequent (12%), and infection connectivity was relatively high (median IBD = 5%), consistent with low endemicity. Chocó exhibited a higher frequency of polyclonal infections (23%) than Córdoba (7%), although the difference was not significant (*P* = 0.300). Most Colombian infections carried double *pvdhfr* (95%) and single *pvdhps* (71%) mutants, but other drug resistance mutations were less prevalent (< 10%). There was no evidence of selection at the *pvaat1* gene, whose *P. falciparum* orthologue has recently been implicated in chloroquine resistance. Global population comparisons identified other putative adaptations. Within the Americas, low-level connectivity was observed between Colombia and Peru, highlighting potential for cross-border spread. Our findings demonstrate the potential of molecular data to inform on infection spread and adaptation.

## Introduction

Colombia aims to eliminate malaria within its borders by 2030 but faces significant challenges. Although the country experienced a 25–50% reduction in malaria cases in the early 2000s, it remains one of the most malaria-endemic nations in South America^1^. In 2020, the combined burden of malaria in Venezuela, Brazil and Colombia accounted for more than 77% of cases in the World Health Organisation (WHO) Americas region^1^. Over seventy thousand malaria cases were reported in Colombia in 2022, ~ 60% of which were caused by *Plasmodium vivax* infection^2^. The parasite forms dormant liver stages (hypnozoites) that reactivate weeks to months after an initial infection to cause relapsing episodes of malaria, and these, in conjunction with persistent low-density blood-stage infections, confer high potential for *P. vivax* spread within and across borders. The threat of *P. vivax* is further compounded by the recent political crisis in neighbouring Venezuela, which has led to a major resurgence of this species in the region^1^. As Colombia progresses toward malaria elimination, vigilant surveillance of the residual *P. vivax* transmission is critical.

*Plasmodium vivax* epidemiology including transmission patterns, relapse dynamics and treatment efficacy remain poorly characterised in Colombia. Clinical trials suggest low rates of *P. vivax* chloroquine resistance, but these studies are not conducted routinely and can be difficult to interpret owing to potential confounding by relapsing infections^3,4^. The impact of antimalarials used to treat the co-endemic *P. falciparum* population on the molecular epidemiology of *P. vivax* in Colombia also remains unclear. To reach the ambitious malaria elimination milestones proposed by the Colombian Ministry of Health, the Pan American Health Organization and the Amazon Network for the Surveillance of Antimalarial Drug Resistance (PAHO/RAVREDA), it is paramount to generate detailed information on how the *P. vivax* populations are evolving and spreading within and across borders.

The inability to maintain *P. vivax* parasites in continuous ex vivo culture has significantly constrained our understanding of the biology and epidemiology of this species; genomic studies offer an alternative approach to generate new insights^5,6^. Although several genomic studies have been conducted on Colombian *P. vivax* isolates, all but one small study, have evaluated the pooled isolates collected across Central and South America^7–9^. Whilst genomic epidemiology investigations of *P. falciparum* in Colombia using identity-by-descent (IBD) based measures have informed on local and cross-border infection connectivity, similar approaches have yet to be applied to the in-depth analysis of in *P. viv*ax^10,11^.

Using 64 Colombian *P. vivax* genomes (doubling the sample size of previous studies), we provide a detailed description of within-host infection relatedness in the two major foci of vivax transmission: Chocó and Córdoba. We explore IBD-based infection connectivity within Colombia and across borders in the Americas, and search for novel adaptations in drug resistance and other functions.

## Methods

### Study sites

**Figure 1:**
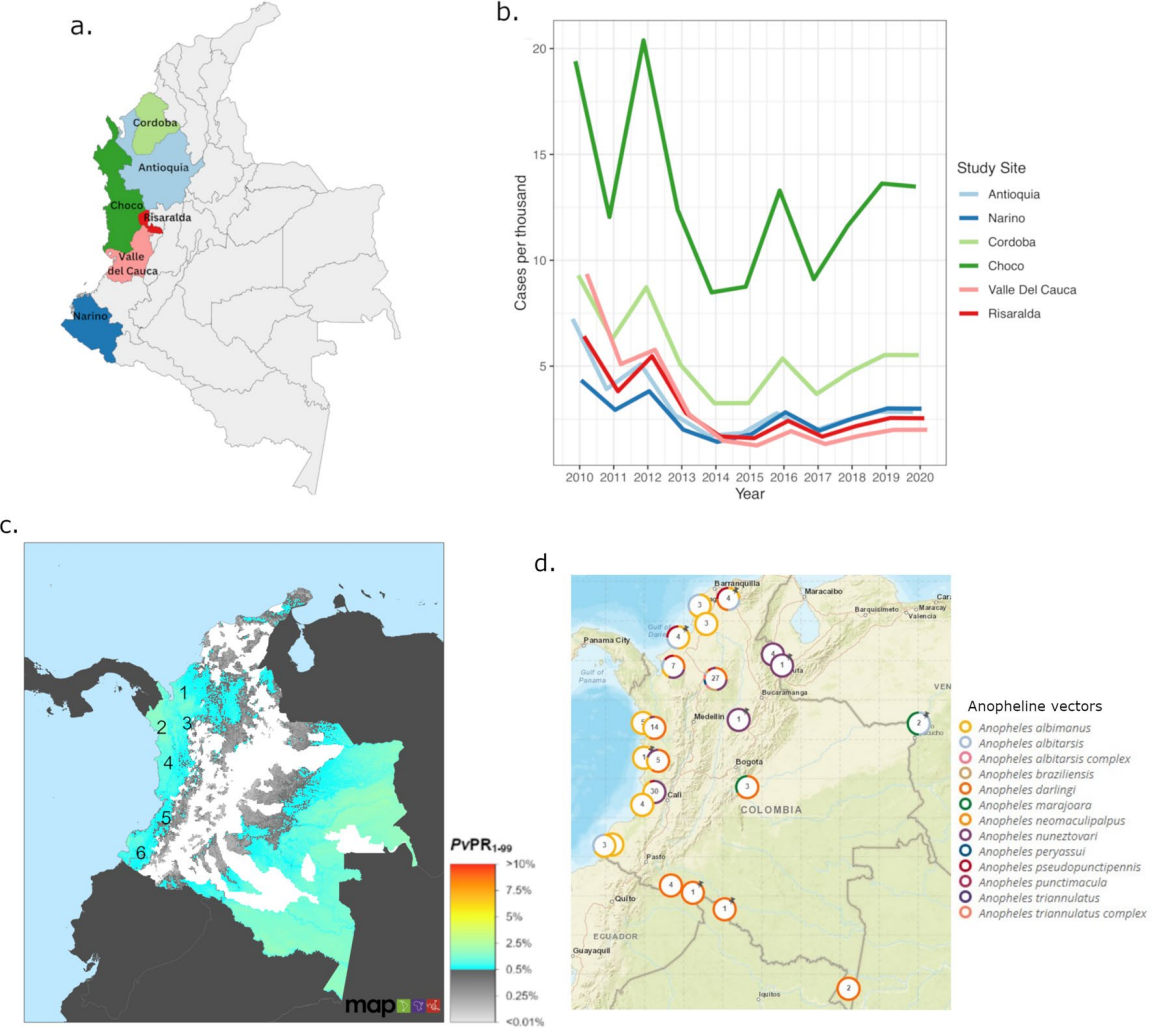
Location and malaria incidence of the study sites. Panel (**a**) presents a map illustrating the locations of the study sites prepared using Canva (https://www.canva.com). Panel (**b**) presents *P. vivax* clinical case numbers between 2010 and 2020 in each of the study sites, using data derived from the Malaria Atlas Project (https://malariaatlas.org/trends/country/COL, accessed September 1st, 2023)^77^. A sharp decline in case numbers was observed between 2010 and 2015, followed by a modest increase in cases and stabilised frequency from 2019. Panel (**c**) presents a map of all-age *P. vivax* prevalence rate (*Pv*PR_1-99_) in Colombia derived from the Malaria Atlas Project (https://malariaatlas.org/trends/country/COL, accessed September 1st, 2023)^77^. The approximate locations of the study sites are indicated with numbers; 1 Córdoba, 2 Chocó, 3 Antioquia, 4 Risaralda, 5 Valle del Cauca and 6 Nariño, illustrating localisation in vivax-endemic regions across the pacific coast. Panel (**d**) provides a map derived from VectorBase illustrating Anopheles species presence in Colombia (vectorbase.org/popbio-map/web, accessed September 1st, 2023)^77^. A range of species were reported in Chocó and Córdoba (major foci of the current study), with geographic heterogeneity observed within the departments, but *An. albimanus*, *An. darlingi* and *An. nuneztovari* were prevalent in both departments.

The Colombian *P. vivax* genomic data were derived from new and previously described patient isolates collected within the framework of clinical studies and cross-sectional surveys conducted between 2013 and 2017. Briefly, published *P. vivax* genomes were obtained from studies undertaken in the departments of Antioquia, Chocó, Córdoba, Nariño, Risaralda, and Valle del Cauca between 2013 and 2017 (Fig. 1a)^8,12,13^. Patients presenting with uncomplicated symptomatic malaria attending health care centres were invited to participate in the studies. *P. vivax* accounts for ~ 50% of malaria infections in the study areas, with an annual parasite incidence (API) during the study period of between 0.14 and 32.6 cases per 1000 population across the sites^14^. Between 2013 and 2017, Chocó presented the highest *P. vivax* API, followed by Cordoba (Fig. 1b). Additional information on the malaria epidemiology of the study sites is presented in Fig. 1c, which illustrates the underlying *P. vivax* prevalence, and Fig. 1d, which illustrates the distribution of *Anopheline* vectors. Colombia has four major transmission areas: a north-western region (encompassing Uraba, Sinu and Bajo Cauca, where Cordoba is located), the western Pacific coast region (including Chocó), the eastern Orinoquia region and the southernmost Amazonian Region^15^. Chocó and Cordoba present similar socio-economic and infrastructure conditions^16,17^. Malaria transmission is mostly peri-urban and high-risk areas are located near to vegetation or mining areas^16,17^. In the Pacific coast region, the main vectors are *An. albimanus, An. nuneztovari* and *An. darlingi*^18,19^. The first line policy for treating *P. vivax* infection in Colombia is chloroquine (CQ) at 10 mg/kg on days 1 and 2 and 5 mg/kg on day 3, and primaquine (PQ) at 0.25 mg/kg for 14 days^1^. Artemether-lumefantrine (AL) is used to treat *P. falciparum* and mixed-species *P. falciparum* and *P. vivax* infections^1^.

To determine cross-border infection across the Americas, and identify signals of selection in Colombia, previously published genomic data from a global collection of *P. vivax* isolates were derived from the Malaria Genomic Epidemiology Network (MalariaGEN) *P. vivax* community project Pv4 data release^13^. Other major populations from the Americas included Brazil, Peru and Mexico. For context in interpreting trends at drug resistance candidates, CQ + PQ is first-line treatment for *P. vivax* in all three countries, whereas *P. falciparum* and mixed-species infections are treated with AL + PQ or artesunate-mefloquine (AS-MQ) + PQ in Brazil, AS-MQ + PQ in Peru, and AL in Mexico^1^.

In addition to the Americas, comparative explorations of candidate drug resistance allele frequencies and signals of selection were undertaken against MalariaGEN Pv4 samples from Thailand and Papua Indonesia. The Thai population was included as a representative of a region with low-grade CQ resistance, while the Papua Indonesian population represented a region with high-grade CQ resistance^20–22^. The samples were collected from symptomatic patients attending outpatient clinics in Tak Province, Thailand (2006–2013) and Mimika district, Papua Indonesia (2011–2014). The frontline treatment for *P. vivax* infection at the time of the enrolments was CQ plus PQ in Thailand and dihydroartemisinin-piperaquine (DHP) plus PQ in Indonesia^1^.

### Sample processing and whole genome sequencing

Genome-wide single nucleotide polymorphisms (SNP) and copy number variants (CNV) data were derived from the MalariaGEN Pv4 release^13^. All Pv4 data originate from *P. vivax*-infected patient blood samples that were subject to paired-end Illumina sequencing, with the latest release reflecting a combination of new and previously described *P. vivax* genomes^13^. All sequence data were subject to the same alignment, variant calling, and genotyping processes. Briefly, human reads were removed by mapping to the human reference genome using *bwa* software, and the remaining reads were mapped to the *P. vivax* P01 v1 reference genome^23,24^. SNPs and small insertions and deletions (indels) were called following GATK Best Practices Workflows^25,26^. The resultant Variant Calling Format (VCF) file describing ~ 4.5 million variants in 1,895 worldwide *P. vivax* genomes is openly accessible on the MalariaGEN website (https://www.malariagen.net/data/open-dataset-plasmodium-vivax-v4.0). The Pv4 dataset also provides information on large CNVs (> 3Kbp) detected using custom Python scripts.

Prior to conducting population genetic analyses, several sample and SNP filtering steps were undertaken on the original Pv4 VCF. Samples were filtered to remove genomes with low sequence coverage, mixed-species, and apparent outliers, leaving 1,031 analysis-ready samples. The analysis-ready samples were classified by geographic region, with listings for Colombia (n = 67), Americas (n = 159), and Americas combined with Thailand and Indonesia (Asia-Americas, n = 479). The Americas dataset comprised samples from Brazil, Colombia, El Salvador, Mexico, Nicaragua, Panama, and Peru. SNP filtering was performed on a VCF comprising the Asia-Americas sample set (n = 479), with country subsetting performed after. Firstly, all genotypes that were supported by less than five reads were re-coded as missing to exclude low confidence genotype calls. Two separate VCF files were then created: i) a VCF comprising candidate drug resistance determinants for analysis of resistance prevalence, and ii) a VCF comprising all (n = 911,901) high-quality bi-allelic SNPs for all other analyses. The 911,901 SNPs were further filtered to exclude monomorphic positions, leaving 430,520 SNPs (~ 47.21% retained). Next, the genotype failure distribution of samples was investigated and SNPs with excess sample failure (> 10% sample fails) were removed. This filtering step was repeated using the SNP failure distribution, removing samples with excess SNP failure, defined as > 15% SNP fails. The final (Asia-Americas) dataset comprised 427,199 SNPs across 457 samples.

### Data analysis

Within-host infection diversity was initially measured using the *F*ws score, applying a threshold *F*ws ≥ 0.95 to classify infections as monoclonal^27,28^. *F*ws scores were derived from the measures provided in the Pv4 open dataset and plotted using ggplot2^13,29^. Within each polyclonal infection, *DEploid* was used to derive the genetic phase of all high frequency clones (clones making up ≥ 10% of the infection)^30,31^ (https://github.com/DEploid-dev/DEploid). Firstly, population level allele frequencies (PLAF) were calculated from monoclonal (n = 56) and polyclonal (n = 8) Colombian samples. Monomorphic SNPs or those with missing data were removed, leaving 26,758 SNPs for analysis. A reference panel was created by first running *DEploid* on the monoclonal samples after marking outlier SNPs using *DEploid* dataExplore.r. Only clones with 99% proportion from each monoclonal sample were included in the reference panel. Haplotypes from these clones were collected and SNPs with missing data were removed, resulting in a reference panel with 56 haplotypes and 23,147 SNPs. The *DEploid-BEST* algorithm was then run on the polyclonal samples with the PLAF and reference panel after marking outlier SNPs, with non-default parameters of sigma = 2.0 and VQSLOD = 0 to generate haplotype estimates of high frequency clones. Using the phased haplotype reconstructions, the pairwise identity by descent (IBD) between the high frequency clones inferred by *DEploid* was measured using *hmmIBD* software, with default parameters^32^. Illustrative representations of the within-host diversity in the polyclonal infections were also prepared by plotting the genome-wide non-reference allele frequency (NRAF). The NRAF plots were created using custom scripts generated with *R* software (www.R-project.org). All other analyses were restricted to monoclonal infections to avoid potential inaccuracies from incorrect phase re-construction.

Population structure and infection relatedness were explored using *ADMIXTURE*, neighbour-joining, and IBD analysis^32–34^. The R-based *ape* package was used to generate distance matrices and build neighbour joining trees to illustrate infection diversity and relatedness^34^. *hmmIBD* was used to calculate IBD between infections^32^. The output from *hmmIBD* (hmm_fract) was used to estimate IBD sharing between samples with plots of infection connectivity at different IBD thresholds prepared using the R-based *igraph* package (https://igraph.org).

The prediction toolbox *snpEff* was run on the drug resistance candidate dataset to annotate the mutations^35^. The results were then enhanced by adding InterPro domain predictions from PlasmoDB^36,37^. A set of known mutations that correlate with drug resistance in *P. vivax* were derived from the literature^38,39^.

Haplotype-based tests to identify genomic regions with evidence of extended haplotype homozygosity (EHH) indicative of recent positive selection were undertaken using *rehh* software^40^. Both the integrated haplotype score (*iHS*) and *Rsb*-based cross-population EHH score were measured. Recommendations for unpolarised data were followed in accordance with the unpolarised nature of the data. A maximum of 10% missing genotypes were allowed, in line with our sample processing methods. The *iHS* was measured in Colombia, and *Rsb* was measured in comparisons between Colombia against each of the populations in the Asia-Americas data set. Prior to running *rehh*, in populations with evidence of large clonal expansions, a single representative sample was used for each clonal cluster to reduce the impact of structure. Clonal clusters were defined as samples sharing ≥ 95% IBD, and the sample with the lowest genotype failure was selected as the representative. In accordance with previous studies, SNP-based *p*-values corresponding with thresholds of *iHS* ≥ 4 and *Rsb* ≥ 5 were considered significant, and candidate signals of selection were defined as regions with a minimum of 3 significant SNPs within a 10 kb window^7,41^.

### Ethics

All patient samples from the MalariaGEN Pv4 dataset were collected with written, informed consent from the patient or a parent or guardian for individuals less than 18 years old^13^. Ethical approval for patient sampling from the newly described study sites in Colombia was provided by the Comité Institucional de Ética de Investigación con Humanos del CIDEIM, Cali, Colombia (reference 08–2015) and the Comité de Bioética Instituto de Investigaciones Médicas Facultad de Medicina Universidad de Antioquia, Antioquia, Colombia (reference BE-IIM): all methods were performed in accordance with the guidelines and regulations of these committees.

## Results

### Genomic data summary

A set of 457 (95%, 457/479) *P. vivax* genomes and 427,199 SNPs were selected based on sample genotyping success rate ≥ 85% and SNP genotyping success rates ≥ 90%. A subset of 142 samples, including 64 Colombian isolates, was selected for analyses within the Americas. The 64 Colombian samples included 36 new isolates from Colombia (detailed in Supplementary Data 1), and 28 previously analysed samples from Colombia^8^. The Colombian samples came from 6 departments (see Fig. 1), but population-level statistics were only detailed on the samples from Chocó (n = 13) and Córdoba (n = 40). Owing to small sample size (n < 10), the samples from Antioquia, Nariño, Risaralda and Valle del Cauca were only included in country-wide population statistics and spatial analyses to identify potential geographic trends.

### Within-host diversity in Colombia

**Figure 2:**
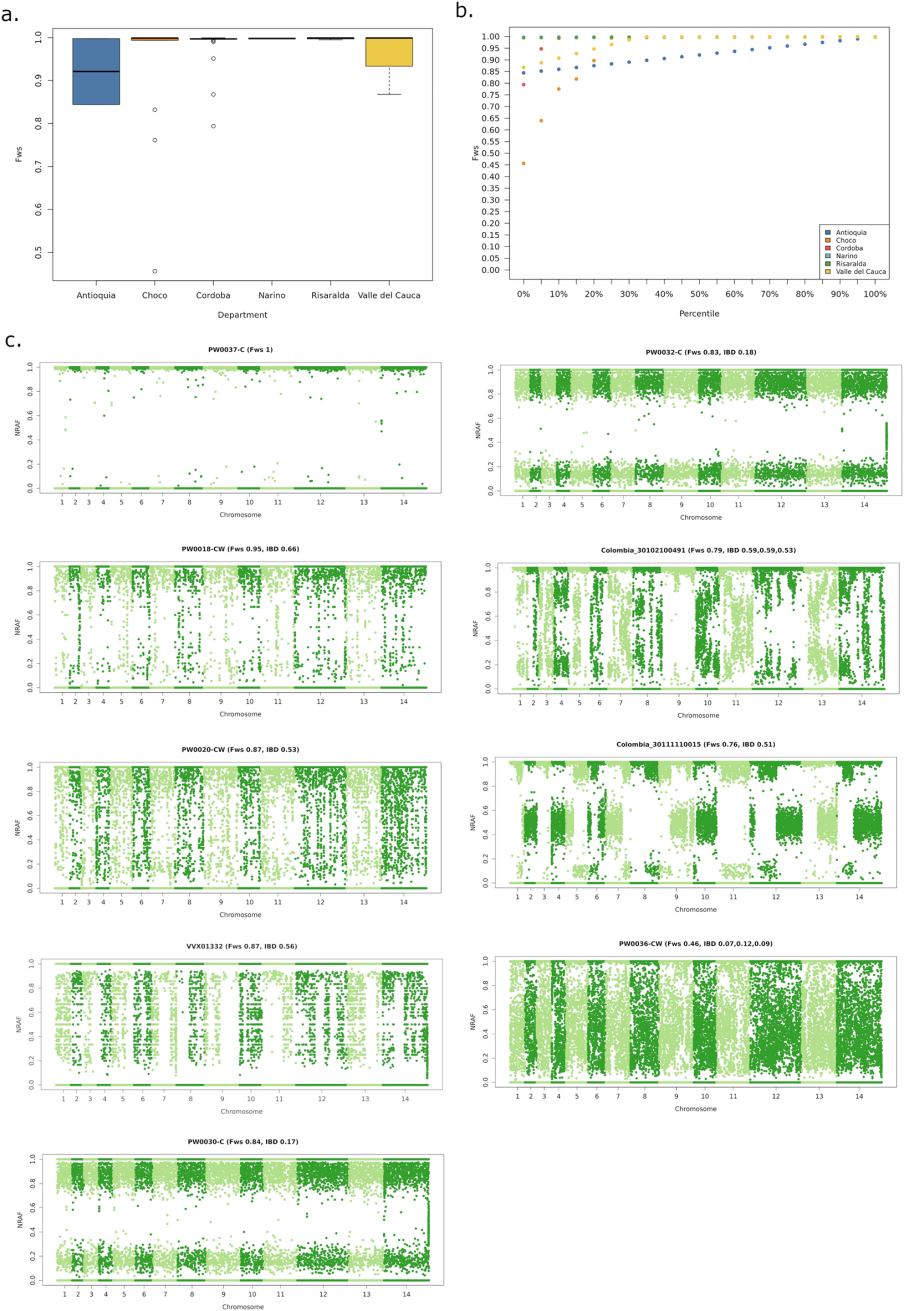
Within-sample infection diversity. Panels (**a**) and (**b**) present box plots and percentile plots, respectively, illustrating the distribution of within-sample F statistic (*F*_WS_) scores in Colombia. Data are presented on all 64 high-quality samples. Panel (**c**) presents Manhattan plots of the non-reference allele frequency (NRAF) in the 8 Colombian infections identified as polyclonal based on *F*_WS_ < 0.97, and 1 monoclonal infection as a baseline reference (PW0037-C). Pairwise measures of identity by descent (IBD) are indicated for pairings of the clones making up 10% or more of the given infection.

Within-sample infection complexity was assessed using the *F*_WS_ score. Initially, the commonly applied threshold of *F*_WS_ ≥ 0.95 was used to define monoclonal infection, but closer inspection of the non-reference allele frequency (NRAF) distribution within infections revealed that an infection with *F*ws = 0.95 (PW0018-C) had evidence of multiple clones (Fig. 2c). A more stringent threshold of *F*_WS_ ≥ 0.97 was therefore used to define monoclonal infection across samples in the Asia-America dataset. Across Colombia, 88% (56/64) infections had *F*_WS_ scores ≥ 0.97. A lower proportion of monoclonal infections was observed in Chocó (77%, 10/13) than Córdoba (93%, 37/40), but the difference was not significant (χ^2^ = 1.074, *p* = 0.300) (Fig. 2a,b, Table 1). As illustrated in the non-reference allele frequency (NRAF) plots presented in Fig. 2c, a range of within-host diversity patterns were observed across the 8 polyclonal infections.

**Table 1:** Summary statistics of within-host infection diversity.

Site	Median *F*_WS_ (range)	% *F*_WS_ ≥ 0.97 (no./No.)	% Polyclonal infections with inter-clone IBD ≥ 0.25*
Chocó	0.999 (0.456–0.999)	77 (10/13)	33 (1/3)
Córdoba	0.997 (0.794–0.999)	93 (37/40)	100 (3/3)
Across Colombia	0.997 (0.456–0.999)	88 (56/64)	63 (5/8)

*Requirement for a minimum of 2 clones with IBD ≥ 0.25 where infections comprise 3 or more clones at ≥ 10% frequency.

In addition to the polyclonal infections from Chocó (n = 3) and Cordoba (n = 3), one polyclonal infection was detected in Antioquia (PW0030-C, two clones with IBD = 0.17) and one in Valle del Cauca (VVX01332, two clones with IBD = 0.56).

DEploid-BEST analysis of the 8 polyclonal samples identified two infections (Colombia_30102100491 from Córdoba and PW0036-CW from Chocó) comprising 3 clones at ≥ 10% frequency. Whilst the 3 Colombia_30102100491 clones appeared to be siblings, with IBD ranging from 0.53 to 0.59, the PW0036-CW clones were more distantly related (IBD range 0.07–0.12) (Fig. 2c). The remaining infections comprised 2 clones at ≥ 10% frequency. In total, 5 (63%) polyclonal infections displayed IBD ≥ 0.25 in pairwise comparisons of at least two clones, suggesting relatedness at the half-sibling level or greater, and likely reflecting co-transmission (single mosquito inoculation) rather than superinfection (multiple inoculations). The remaining 3 infections exhibited IBD ranging from 0.07 to 0.18 (Fig. 2c). Two samples (PW0030-C and PW0032-C) displayed highly uniform NRAFs across all SNPs, consistent with highly divergent major and minor clones. In these two infections, the uniform NRAF profiles enabled accurate approximation of phase in the major (highest frequency) clones by selecting the predominant alleles at each SNP. The inclusion of the phased major clones in each of PW0030-C and PW0032-C yielded 2 additional monoclonal samples for analysis in Colombia, bringing the sample size to 58.

### Infection connectivity within Colombia

**Figure 3:**
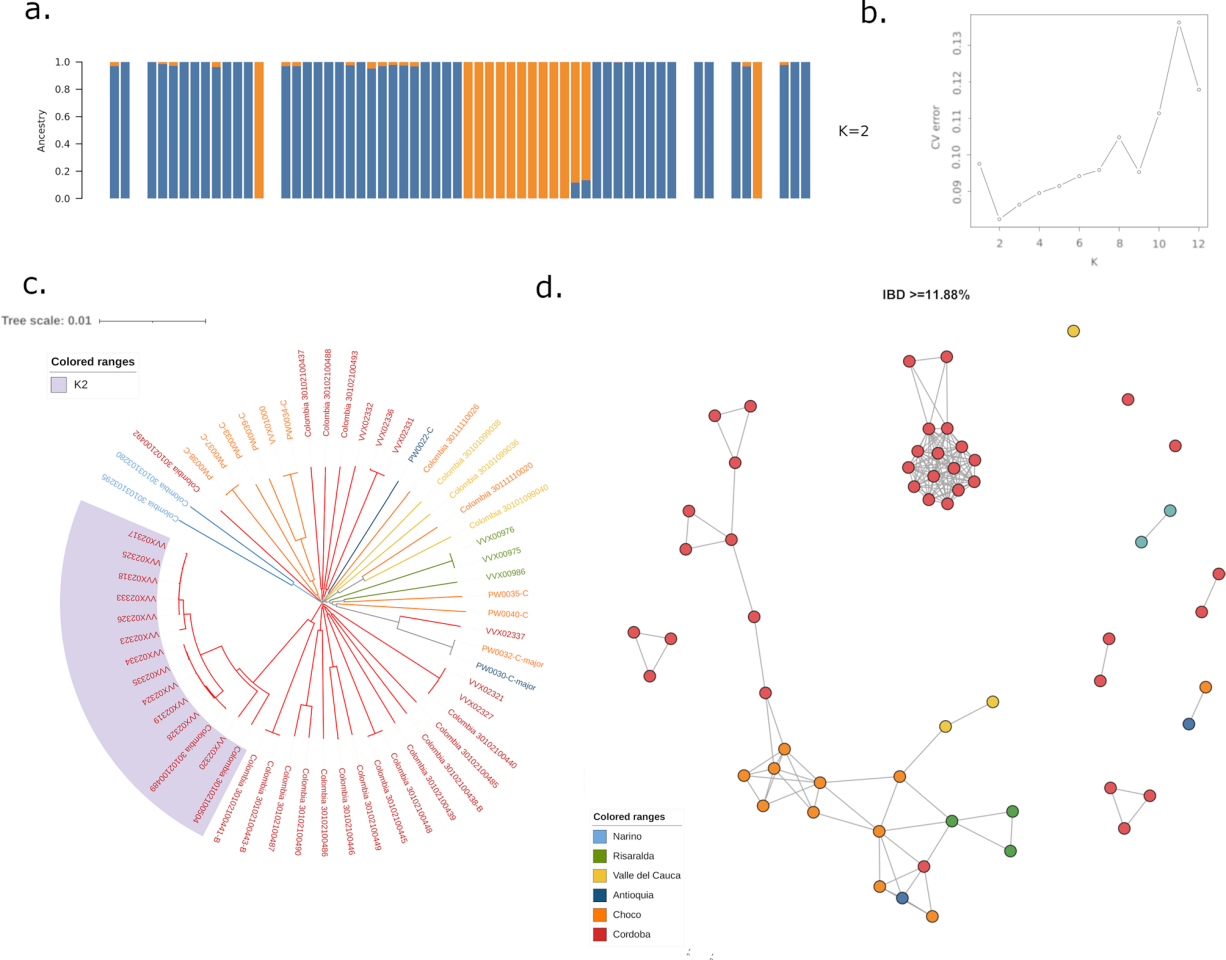
Infection relatedness within Colombia. Panels (**a**) and (**b**) present ADMIXTURE plots, illustrating the CV error at different values of K (panel **b**) and the proportionate ancestry of samples (vertical bars) to each of the two subpopulations at K = 2 (panel **a**). The lowest CV error was observed at K = 2; at this K, the majority of infections across Colombia had predominant ancestry to K1 (in blue), but a sizable proportion of samples from Córdoba had predominant ancestry to K2 (orange). Panels (**c**) and (**d**) illustrate a rooted neighbour-joining (NJ) tree and identity by descent (IBD)-based cluster network, respectively. Both NJ and IBD analyses demonstrate the high relatedness amongst the K2 infections, indicative of clonal expansion dynamics. The cluster network illustrates connections between infections sharing a minimum ~ 12% genomic IBD. All plots present monoclonal Colombian infections only.

Several methods were used to investigate the structure and relatedness between infections in Colombia. Using *ADMIXTURE* analysis, the lowest CV error (0.082) was observed at K = 2, but minimal differences were observed between K = 1 and 7 (range 0.082–0.098) (Fig. 3b). At K = 2, 76% (44/58) samples demonstrated predominant ancestry (> 75% ancestry) to the major subpopulation (K1), and 24% (14/58) to subpopulation K2 (Fig. 3a). Majority of K2 infections derived from Córdoba, making up 32% (12/37) isolates in this department, but one K2 infection was observed in Chocó (9%, 1/11) and one in Risaralda (33%, 1/3). To investigate potential temporal determinants of the observed structure, we added information on year of collection to the samples from Chocó and Córdoba (Supplementary Fig. 1). Although the K2 subpopulation was observed in Córdoba in 2013 and 2016, it was more common in the later time point in both Chocó (100% K2 in 2016) and Córdoba (59% K2 in 2016).

Neighbour-joining (NJ) trees were prepared to visually inspect patterns of relatedness amongst the infections and further explore the substructure identified with *ADMIXTURE* (Fig. 3c). NJ analysis revealed multiple occurrences of infections with identical or near-identical genomes, which we refer to hereafter as clonal clusters. In total, 29 (50%) Colombian infections shared identical or near-identical genomes with at least one other infection. The largest clonal cluster comprised 6 infections, all deriving from Córdoba, but clonal clusters were also observed in other sites. There was only one observation of clone pairs across different sites, namely PW0030-C and PW0032-C from Antioquia and Chocó, respectively. Inspection of the infections by the ADMIXTURE classifications revealed that the K2 subpopulation comprised highly related infections, including the two largest clonal clusters, reflecting relatively more unstable transmission than in the more diverse K1 subpopulation. Investigation of temporal trends revealed that 3 clonal clusters comprised infections presenting in the same site but > 1 year apart (up to 3 years apart), reflecting highly persistent strains (Supplementary Fig. 1).

Since malaria parasites are recombining organisms, NJ analysis can potentially miss recent connectivity between infections where outcrossing has taken place. We therefore further investigated the connectivity between infections using measures of IBD that account for recombination. At IBD thresholds of ~ 0.12 (12%) and greater, reflecting relatively recent common ancestry (quarter-siblings and closer relatives), two large infection networks were observed (Fig. 3d). One cluster was spatially diverse, comprising 42 samples deriving from all sites except Nariño, indicative of shared reservoirs between different regions of Colombia. The second, tighter, cluster comprised 16 samples, all deriving from Córdoba and reflecting the highly related K2 infections. At increasing IBD thresholds, the spatially diverse cluster rapidly broke down, but most K2 infections exhibited connectivity > 0.5 (siblings) (Supplementary Fig. 2). Inspection of temporal trends in IBD revealed multiple closely connected (≥ 12% IBD) infection pairs that were separated by > 1 year (n = 28 samples amongst these connections), confirming the potential for high temporal stability of infection lineages (Supplementary Fig. 2).

### Infection connectivity between Colombia and neighbouring countries

**Figure 4:**
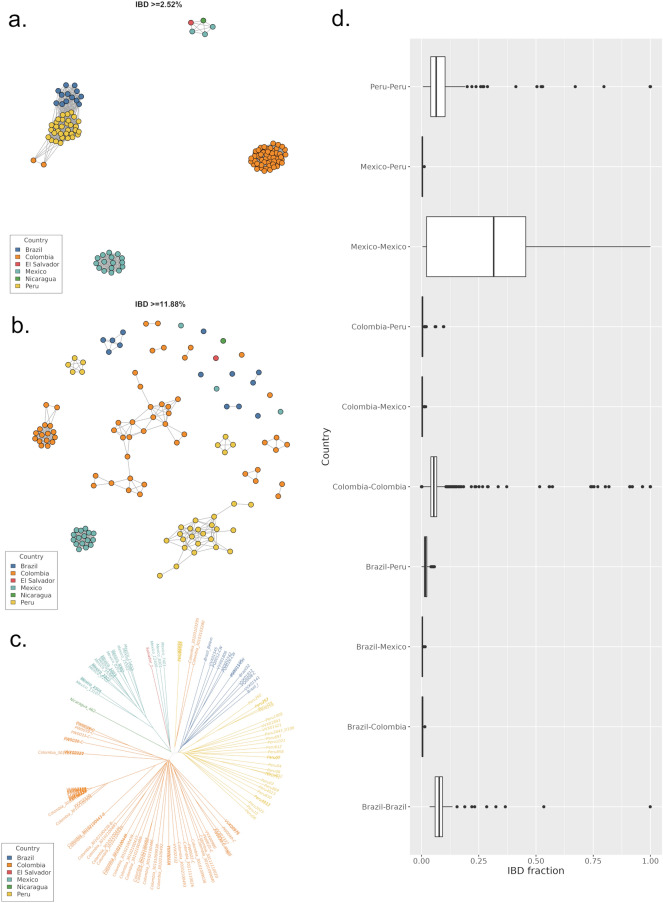
Infection connectivity between Colombia and other populations in Central and South America. Panels (**a**) and (**b**) present IBD plots at minimum thresholds of ~ 2.5 and 12%, respectively, illustrating distant connectivity between several countries but no evidence of cousins or closer relatives across borders. Panel (**c**) presents an unrooted neighbour-joining (NJ) tree confirming the patterns observed with the IBD analysis, including the clustering of two Colombian infections (Colombia_30103103295 and Colombia_30103103280) with several Peruvian infections via a distant shared lineage. Panel (**d**) presents box plots illustrating the distribution of pairwise IBD within and between countries. As observed with the IBD and NJ analyses, IBD is generally greater within than between countries, with the greatest relatedness (lowest outcrossing) observed within Mexico. All plots present monoclonal samples from the Americas dataset only.

To further investigate connectivity, we measured IBD between monoclonal *P. vivax* infections from Colombia and other American countries in the Pv4 dataset; Brazil (n = 13), El Salvador (n = 1), Mexico (n = 19), Nicaragua (n = 1) and Peru (n = 32). The IBD between all populations was low, with no inter-country connectivity observed at thresholds > 10% (Fig. 4a,b). At the relatively low IBD threshold of 2.5%, two infections from Nariño (2013), in the south of Colombia, (Colombia_30103103295 and Colombia_30103103280) demonstrated connectivity with four isolates from Sullana, northern Peru collected in 2011. For context, summary statistics on the IBD within and between countries were prepared, demonstrating that the ~ 2.5% IBD between the 2 Colombian and 4 Peruvian isolates was lower than the median and mean IBDs within Colombia (4% and 5% respectively) and Peru (4% and 7% respectively) (Fig. 4d, Supplementary Table 1).

The accuracy of IBD estimation is dependent in part on the available data on allele frequency, and thus confounded by limited sample size in several countries, as well as the potential constraints in pooling infections across multiple countries. To account for this, NJ analysis was conducted on all infections from Central and South America and confirmed the closer relatedness between isolates Colombia_30103103295 and Colombia_30103103280 with Peruvian infections than with other Colombian infections, potentially reflecting distant cross-border infection spread (Fig. 4c).

NJ analysis was also performed on the Asia-Americas dataset, confirming previously described divergence between infections from the Americas and the Asia–Pacific region (Thailand and Indonesia), as well as the relatively higher diversity in the latter populations (Supplementary Fig. 3)^13^. IBD-based connectivity is also presented on the global dataset, although caution is advised in interpretation owing to biases in global allele frequency derivations (Supplementary Fig. 3).

### Prevalence of drug resistance candidates in Colombia

**Figure 5:**
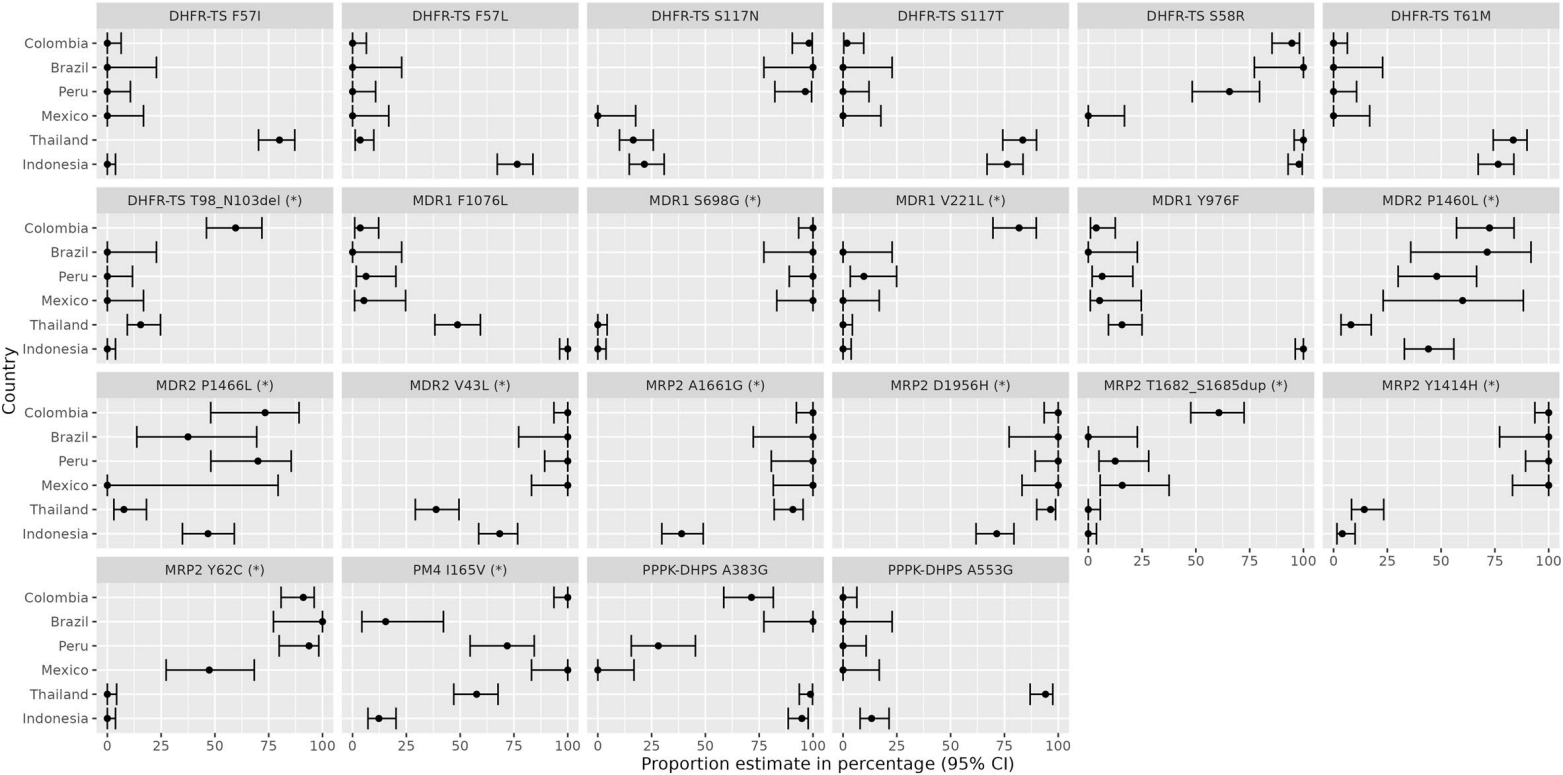
Amino acid frequencies at selected *P. vivax* drug resistance candidates. Each panel presents proportions and corresponding 95% confidence intervals (CIs) for the given amino acid changes. Data is provided on variants that have i) previously been associated with drug resistance or ii) are non-synonymous variants located in orthologues of *P. falciparum* drug resistance-associated genes and exhibit substantial differences (non-overlapping CIs) in proportion between Colombia and at least one other population; the latter class of variants are denoted with (*). For variants that have previously been associated with clinical or ex vivo resistance, frequencies reflect the drug-resistant amino acid.

The prevalence of several candidate drug-resistance variants that have previously been associated with clinical or ex vivo antimalarial drug resistance in *P. vivax* was determined in Chocó, Córdoba and Colombia-wide (Table 2) ^39^. Country-level trends between Colombia relative to Peru, Brazil, Mexico, Thailand, and Indonesia are also illustrated in Fig. 5. The multidrug resistance 1 (*pvmdr1*) Y976F variant, which is a minor modulator of CQ resistance^42^, exhibited low prevalence in Chocó (20% (2/10)) and was absent in Córdoba (0/37), with only 4% prevalence across Colombia (2/55). At the country level, the Y976F variant was most prevalent in Indonesia. The F1076L variant, which has also been proposed as a modulator of CQ resistance^42^, exhibited equally low prevalence in Chocó (20% (2/10)), Córdoba (0% (0/38)) and across Colombia (4% (2/57)). Similar trends of low F1076L prevalence were observed in other American populations, but the variant was prevalent in Thailand and Indonesia. A range of mutations in the dihydrofolate reductase (*pvdhfr*) and dihydropteroate synthase (*pvdhps*) genes have been associated with antifolate resistance^43^. The most common *pvdhfr* variants observed in Colombia were the S58R and S117N mutations, giving rise to double mutants at frequencies of 80% (8/10) in Chocó, 100% (36/36) in Córdoba and 95% (52/55) across Colombia. No triple or quadruple *pvdhfr* mutants were observed in Colombia (0% (0/55)). At the country level, a range of patterns were observed at the *pvdhfr* locus, including higher prevalence of the F57I, F57L, S117T and T61M mutations in Asian relative to Central or South American populations. At the *pvdhps* locus, the A383G mutation was prevalent in Chocó (100% (10/10)), Córdoba (66% (25/38)) and across Colombia (72% (41/57)). No *pvdhps* A553G mutations were observed in Colombia (0% (0/57)). In other populations, the *pvdhps* A383G mutation was also prevalent in Brazil, Thailand and Indonesia, and the *pvdhps* A553G mutation was also infrequent in all populations except Thailand. None of the isolates had the *pvmdr1* copy number amplification associated with mefloquine resistance, but this variant analysis was restricted to only 22 Colombian samples that exhibited sufficient coverage^44,45^.

**Table 2:** Prevalence of drug resistance markers in Colombia.

Gene	Chr	Position	Mutation	Drug	Freq, % (no./No.)	Freq, % (no./No.)	Freq, % (no./No.)
					Colombia	Chocó	Córdoba
*pvmdr1*	10	479908	F1076L	CQ	4 (2/56)	20 (2/10)	0 (0/37)
*pvmdr1*	10	480207	Y976F	CQ, AQ + SP	4 (2/54)	20 (2/10)	0 (0/36)
*pvmdr1*	10	CNV	≥ 2 copies	MQ	0 (0/22)	0 (0/7)	0 (0/15)
*pvdhfr*	5	1077530; 1077532	F57L/I	Antifolate, AQ + SP	0 (0/56)	0 (0/10)	0 (0/37)
*pvdhfr*	5	1077533; 1077534; 1077535	S58R	Antifolate, AQ + SP	95 (53/56)	80 (8/10)	100 (37/37)
*pvdhfr*	5	1077543	T61M	Antifolate, AQ + SP	0 (0/56)	0 (0/10)	0 (0/37)
*pvdhfr*	5	1077711	S117N/T	Antifolate, AQ + SP	100 (55/55)	100 (10/10)	100 (36/36)
*pvdhfr*	…	…	Single mutant	Antifolate, AQ + SP	5 (3/55)	20 (2/10)	0 (0/36)
*pvdhfr*	…	…	Double mutant	Antifolate, AQ + SP	95 (52/55)	80 (8/10)	100 (36/36)
*pvdhfr*	…	…	Triple mutant	Antifolate, AQ + SP	0 (0/55)	0 (0/10)	0 (0/36)
*pvdhfr*	…	…	Quadruple mutant	Antifolate, AQ + SP	0 (0/55)	0 (0/10)	0 (0/36)
*pvdhps*	14	1270401	A553G	Antifolate	0 (0/56)	0 (0/10)	0 (0/37)
*pvdhps*	14	1270911	A383G	Antifolate	71 (40/56)	100 (10/10)	65 (24/37)

Prevalence of drug resistance markers that have been associated with clinical or ex vivo drug resistance in *P. vivax.* Mutation prevalence was calculated with homozygous calls only. Abbreviations: CNV, copy number variation; AQ, amodiaquine; CQ, chloroquine; MQ, mefloquine; SP, sulfadoxine-pyrimethamine. Gene identifiers; *pvmdr1* (PVP01_1010900), *pvdhfr* (PVP01_0526600), *pvdhps* (PVP01_1429500).

For reference purposes, the prevalence of other nonsynonymous variants in *pvmdr1*, *pvdhps*, *pvdhfr* and orthologues of several genes implicated in drug resistance in *P. falciparum* is provided for Colombia and the comparator populations in Supplementary Data 2. The orthologues include *pvaat1* (amino acid transporter 1), *pvcrt-o* (chloroquine resistance transporter), *plasmepsin IV*, *pvmrp1* and *pvmrp2* (multidrug resistance–associated proteins 1 and 2), and *pvmdr2* (multidrug resistance protein 2). Notable mutations that exhibited substantial differences in proportion between Colombia and at least one other population are illustrated in Fig. 5 alongside the above-described (known) drug resistance candidates. Substantial differences were observed between Colombia and all other populations in the proportion of *pvdhfr* T98/N103del and *pvmrp2* T1682/S168dup variants. Other variants exhibited regional trends, with differences in proportion between Central and South American relative to the Asian populations observed at *pvmdr1* S698G, *pvmdr1* V221L, *pvmdr2* V43L and *pvmrp2* Y144H. A spectrum of other population trends was observed in the proportions of the *pvaat1* A526dup, *pvaat1* G41S, *pvaat1* R19L, *pvmdr2* P1460L, *pvmdr2* 1466L, *pvmrp2* A1661G, *pvmrp2* D1956H, *pvmrp2* Y62C and *plasmepsin IV* I165V variants.

### Genome-wide scans to identify new adaptations in Colombia

**Figure 6:**
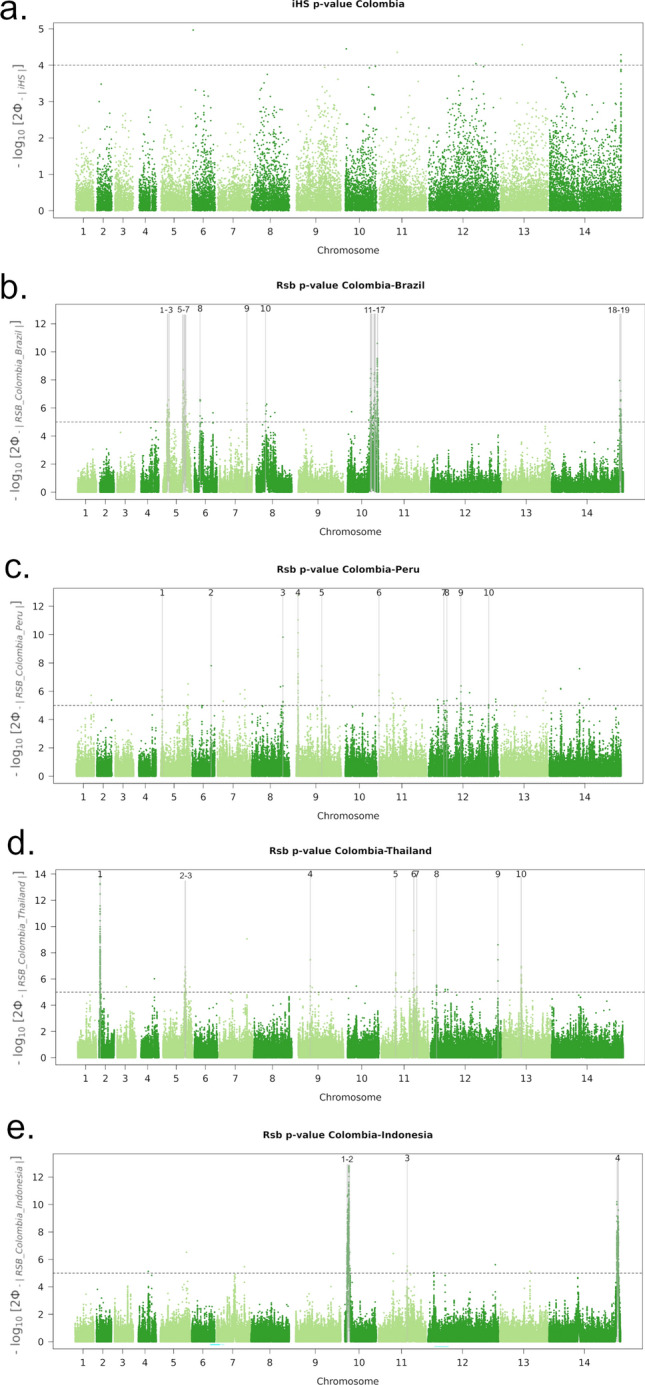
Genome-wide scans of iHS and Rsb-based extended haplotype homozygosity in Colombia. Panel (**a**) presents a Manhattan plot of the iHS −log10(p) in Colombia. The dashed black line demarks a fixed significance threshold of −log10(p) = 4. There were no signals supported by a minimum of 3 single-nucleotide polymorphisms (SNPs) above the threshold. Panels (**b**)–(**e**) present Manhattan plots of the Rsb −log10(p) for the given populations. The dashed black lines demark a fixed significance threshold of −log10(p) = 5: signals supported by a minimum of 3 single-nucleotide polymorphisms (SNPs) above the threshold within 10 kb of one another are demarked with vertical dashed grey lines and numbered. Signals associated with extended haplotypes in Colombia and their putative genetic drivers include: *pvmsp1* (PVP01_0728900) in region 9 in the Brazilian comparison (panel b); 1-acyl-sn-glycerol-3-phosphate acyltransferase (PVP01_1262700) in region 10 in the Peruvian comparison (panel **c**); pyridoxine biosynthesis protein PDX2 (PVP01_0916800) in region 4 in the Thai comparison (panel **d**); a conserved *Plasmodium* protein with unknown function (PVP01_1115800) in region 5 (panel **d**); an oligomeric Golgi complex subunit 4 protein (PVP01_1133300) in region 6 (panel **d**); 6-cysteine proteins P12 (PVP01_1136400) and P47 (PVP01_1208000) in regions 7 and 8 (panel **d**); a metacaspase-2 (PVP01_1268600) in region 9 (panel d); and a PIMMS43 protein (PVP01_1129500) in region 3 in the Indonesian comparison (panel **e**). Further details are provided in Supplementary Table 4.

Evidence of new candidates of drug resistance and other adaptive mechanisms were explored by identifying genomic regions with evidence of relatively extended haplotype homozygosity (EHH) in Colombia. The integrated haplotype score (*iHS*) was applied to the Colombian dataset, and *Rsb*-based cross-population EHH was assessed in Colombia relative to each of the populations that have a minimum of 10 samples. Clonal clusters were represented by a single infection to reduce potential confounding by population structure, producing the following analysis sets; Colombia (n = 39), Brazil (n = 12), Peru (n = 26), Mexico (n = 17), Thailand (n = 85) and Indonesia (n = 98). A false discovery rate (FDR) significance threshold of 0.05 and fixed thresholds of -log10(*p*) = 4 for the *iHS* and 5 for *Rsb* were explored, revealing a high overlap in signal detection with the *iHS* (100%, 0/0) and *Rsb* (64%, 28/44). The fixed thresholds were therefore selected for defining signals to facilitate more direct comparisons with previous studies that have used the same thresholds^7,41^. No significant signals of selection were observed with the *iHS* analysis in Colombia, potentially reflecting constrained statistical power of this method to detect signals when sample size is modest (Fig. 6a, Supplementary Table 2).

Cross-population analyses revealed 19 regions with evidence of differential selection between Colombia and Brazil (Fig. 6b). One previously described signal appeared to be under positive selection in Colombia and encompassed *pvmsp1*, encoding a merozoite surface protein, a promising vaccine candidate that is involved in merozoite formation, and red blood cell invasion and egress^46,47^. Comparisons of Colombia relative to Peru identified 10 signals, one of which was under positive selection in Colombia and contains a 1-acyl-sn-glycerol-3-phosphate acyltransferase gene, putatively involved in cell membrane formation (Fig. 6c) ^48,49^. Even after excluding clonal clusters, there was extensive population structure in Mexican isolates, and thus signals of cross-population EHH against this population were not explored. Outside of the Americas, comparisons against Thailand revealed 10 signals of differential selection, including 5 regions under positive selection in Colombia (Fig. 6d). The Colombian signals reflect a variety of putative drivers including a pyridoxine biosynthesis protein PDX2, a conserved oligomeric Golgi complex subunit 4 protein, 6-cysteine proteins P12 and P47, whose *P. falciparum* orthologs are involved in host immune evasion, a metacaspase-2, whose ortholog has a role in malaria transmission, and a conserved *Plasmodium* protein with unknown function^50,51^. Four signals were observed against Indonesia, with one region representing selection within Colombia (Fig. 6e). This signal comprises a PIMMS43 protein, implicated in immune evasion in *P. falciparum* and *P. berghei*^52^.

In addition to SNPs, we sought evidence of adaptations mediated by large (> 3 kb) tandem duplications provided in the MalariaGEN Pv4 data release but found no evidence of duplications in Colombia (Supplementary Table 3)^13^. However, sample size may have constrained evaluation; for example, there were only 23 samples with suitable read depth at the commonly observed *pvdbp1* duplication.

## Discussion

Our large genomic epidemiology investigation of *P. vivax* in Colombia, demonstrates patterns of within-host and population diversity consistent with low endemicity between 2013 and 2017. Although this level of endemicity is conducive to timely *P. vivax* elimination, there is evidence of low-level shared reservoirs of infection with neighbouring countries, and presence of known drug resistance-associated variants as well as several potential new adaptations. Further details on the observed genetic patterns and their implications for *P. vivax* surveillance and treatment efficacy are discussed.

A major concern for the Colombian National Malaria Control Program (NMCP) is whether local interventions are effective in reducing parasite transmission. Whilst traditional entomological and parasitological measures of infection prevalence and incidence are critical, asymptomatic and subpatent reservoirs are impossible to capture with these approaches. The dormant liver stages of *P. vivax* further complicate measures of the burden of *P. vivax*^53^. Parasite population genetic features provide useful insights on local transmission to complement more traditional measures. For instance, high within-host infection diversity is one such feature, generally reflecting high endemicity^6,53^. Our finding that polyclonal infections occurred in only 12% (8/64) of Colombian isolates aligns with low rates of *P. vivax* transmission, comparable to Malaysia (16% polyclonal infections) during its malaria pre-elimination phase^54^. To put these figures in context, high transmission regions of Papua Indonesia, Papua New Guinea, the Greater-Mekong subregion, and Ethiopia report between 30 and 60% polyclonal infections^7,8,41,55–57^. Other regions of the Americas, including areas of neighbouring Brazil and Peru also exhibit lower polyclonality (13–16%) than the Asia–Pacific region^47^. We also observed that over 60% of the polyclonal infections in Colombia comprised closely related clones such as siblings or half-siblings (> 25% genomic IBD), suggesting recent shared parentage. Parasites with recent shared parentage are more likely to arise from a single mosquito inoculum (i.e., co-transmission of the different clones) than multiple inoculums (i.e., superinfection)^30,58^: under this assumption, our results infer a higher frequency of co-transmission than superinfection events in Colombia, as might be expected as transmission declines. However, using similar methods, a study in a moderately high transmission region of Ethiopia reported a similar frequency (57%) of co-transmission events^41^. With limited information from other endemic regions, it’s unclear the degree to which reactivated hypnozoites, as opposed to reinfections, determine within-infection relatedness patterns in *P. vivax*. Further research in this area using genome phasing approaches or single cell sequencing, will enable a more contextualised view of the relationship between within-host relatedness and *P. vivax* endemicity^59^. Our study provides baseline data for future comparisons to evaluate the ongoing efficacy of interventions in Colombia.

A wide variety of ecological patterns and associated malaria epidemiology have been described in Colombia, highlighting the importance of subnational interventions adapted to local needs^1,14^. Our investigations of within-host diversity at the departmental scale found modest evidence of heterogeneity between sites. Chocó has historically harboured high levels of malaria and is one of the priority areas for malaria elimination in Colombia. Over 60% (2/3 with within-host IBD < 0.25) of the polyclonal infections in Chocó appeared to reflect superinfections in contrast to 0% (0/3) in Córdoba. Chocó also presented a higher frequency of polyclonal infections (23%) than Córdoba (7%), but the sample size in Chocó was constrained (n = 13) and the difference was not statistically significant. Nonetheless, these trends call for ongoing monitoring of transmission reduction efficacy in Chocó. Several factors may explain the observed transmission patterns in Chocó, including the local *Anopheline* vectors. Typically, *An. albimanus, An. nuneztovari* and *An. darlingi* are prevalent, but their frequencies may change with seasonal variation^18,19^. Whilst our study was moderately granular in spatial scale, our findings highlight the potential for genetic epidemiology approaches using more high-throughput methods such as barcode genotyping to capture important infection dynamics.

Population structure and relatedness provide an additional genetic measure yielding insights into local malaria transmission dynamics^53,60^. A notable genetic feature in Colombia was the high frequency of closely related infections, including the large K2 cluster (n = 14 infections with IBD ≥ 50%) observed in Córdoba. This pattern indicates a high degree of inbreeding and has been observed in other low endemic *P. vivax* populations, including Malaysia and Panama as infection prevalence declined and opportunities for outcrossing diminished^54,61^. Similar patterns have also been described in the *P. falciparum* population in Colombia during a period when cases were declining^10,62^. The high degree of inbreeding, particularly in Córdoba, is therefore promising regarding Colombia’s goals of achieving malaria elimination certification. However, our data is from 2013–15 and ongoing surveillance will be critical to avoid resurgence from highly resilient or adaptive strains. Parasite populations with low levels of outcrossing may be more amenable to the emergence of drug-resistant malaria strains^63^. Infrequent outcrossing is favourable when supporting variants are required to overcome fitness costs of drug resistance-conferring mutations. Our study and similar *P. falciparum* studies in Colombia demonstrate a high potential for infection lineages to persist over multiple years in line with infrequent superinfection and hence infrequent outcrossing^62^. *P. vivax* studies in Malaysia and Panama have reported similar trends, with infections persisting up to a decade^54,61^.

Surveillance of *P. vivax* drug resistance is challenged by limited information on the molecular markers^38^. Amongst the few described markers of resistance, we found no evidence of the *pvmdr1* copy number duplication (0% across Colombia) that has been associated with mefloquine resistance, suggesting that it may be a suitable alternative to chloroquine (as a partner drug in artemisinin combination therapies, ACTs)^44,45^. However, the number of samples in our study that were suitable for copy number evaluation was constrained (n = 22, 39%) and hence further surveillance is warranted. Co-endemic *P. falciparum* populations can potentially give insights into the extent of local drug pressure on *P. vivax*. Investigations of *P. falciparum* infections collected at a similar time and location (enrolments in the Pacific coast and Cauca River regions in 2015) identified ~ 7% prevalence of *pfmdr1* copy number amplifications^64^. The selective pressure on *pfmdr1* justifies continued close surveillance of *pvmdr1* amplification in the *P. vivax* population, which may receive inadvertent drug pressure.

Although sulfadoxine and pyrimethamine (SP) are not currently recommended as first-line policy for any malaria species in Colombia, SP has been used to treat *P. falciparum* in the past, in combination with CQ (from 1981 to 1998) and AQ (from 1998 to 2008). In 2008, policy was changed to ACTs owing to widespread therapeutic failures^65,66^. As SP has been shown to have a positive impact on infant birth weight when used in intermittent preventive treatment in pregnancy (IPTp), even to some degree when *P. falciparum* SP resistance variants are present, there is a potential public health application for SP^67^. Our results show evidence of reduced SP efficacy in *P. vivax* in Colombia. Frequencies of the *pvdhfr* S58R + S117N double mutant ranged from 80 to 100%, and the *pvdhps* A383G mutant ranged from 66 to 100% across Colombia. However, there were no triple or quadruple *pvdhfr* mutants and no double *pvdhps* mutants, suggesting that full-grade SP resistance is not common. Previous studies from earlier years have also documented moderate to high prevalence of the *pvdhfr* S58R + S117N double mutants in Colombia^43,68^. Similar patterns have been reported in *P. falciparum* in Colombia, with a 2018 study in Chocó reporting 100% double *pfdhfr* mutants, and 43% single *pfdhps* mutants^69^. Our genomic data also showed evidence of similar trends in *P. vivax* in neighbouring Brazil and Peru, and no SP resistance variants in Mexico. Other studies have also documented the lack of *pvdhfr* mutations in Mexico or Nicaragua^70^. In a separate study from French Guyana, several *pvdhfr* and *pvdhps* mutations were present at very high prevalence, but appear not to have spread to other parts of the Americas^71^. In contrast to the Americas (aside from French Guyana), Thailand and Indonesia exhibited high mutation rates, potentially reflecting differences in drug use between these regions. In addition to the known SP resistance candidates, we identified a *pvdhfr* T98_N108del that was prevalent (~ 60%) in Colombia but absent in the other populations. The functional impact of the *pvdhfr* T98_N108del remains unclear but its high prevalence in Colombia justifies further investigation. Further investigation is also required to understand the utility of the antifolates in IPTp for *P. vivax* amidst different *pvdhfr* and *pvdhps* backgrounds.

Chloroquine (CQ) remains the frontline treatment for blood-stage *P. vivax* infections in Colombia, but no validated markers of clinical efficacy have been identified for this species^38^. Following evidence of clinical failures, CQ was removed from *P. falciparum* treatment policy in Colombia in 2008, but the resistance-conferring *pfcrt* K76T mutation remains prevalent^69^. In addition to *pfcrt*, hard selective sweeps postulated to reflect CQ pressure have also been reported at *pfaat1* in the Pacific Coast of Colombia^10^. In contrast to *P. falciparum*, clinical surveys of CQ efficacy against *P. vivax* have shown failure rates below 3% in Colombia^72^. There was no evidence of selection in either of *pvcrt-o* or *pvaat1* in our study. For reference purposes, we documented the frequencies of the *pvmdr1* Y976F and F1076L mutations that have been reported to be minor determinants of CQ resistance in *P. vivax*, finding 0–20% frequency across Colombia. In other studies, in the Americas, the prevalence of *pvmdr1* Y976F and F1076L alleles ranged from 0% prevalence in Mexico, 4–13% in Peru, and 62–100% in Nicaragua^73,74^. For context, the frequencies in Colombia are comparable to Thailand, where ~ 10% infections carried the Y976F mutation, and < 10% CQ failure rate was reported^20^. In contrast, 100% of the Papua Indonesian infections carried the Y976F mutation; a population where > 60% chloroquine failure was reported by day 28 in the early 2000s^22^. However, the interpretation of these findings in the context of CQ’s efficacy in Colombia is constrained and will require validated markers.

Using information on orthologous genes involved in drug resistance in *P. falciparum,* and hypothesis-free methods to detect signals of selection, we identified several candidate markers of resistance and other adaptations. The candidates include non-synonymous variants in orthology-based drug resistance candidates *pvmdr2*, *pvmrp2*, and *plasmepsin IV* noted for substantial inter-population differences in frequency. Using haplotype-based signals of selection, putative adaptations were identified in genes involved in a range of other functions including vitamin B6 synthesis (pyridoxine biosynthesis protein PDX2), intracellular transport (oligomeric Golgi complex subunit 4), immune evasion (6-cysteine proteases P12 and P47, and PIMMS43), and malaria transmission (metacaspase-2)^50–52^. However, signals of extended haplotype homozygosity can be complex to decipher, and these candidates will require further exploration in functional studies.

As Colombia invests efforts towards malaria elimination, detection of imported cases and other key reservoirs will be critical to avoid resurgence. Imported malaria is a particular challenge for *P. vivax,* where the dormant liver stages and highly persistent subpatent and asymptomatic infections can promote infection spread and confound the accuracy of travel histories. Our study identified two infections from Nariño, Colombia, that exhibited higher genetic relatedness to infections from Sullana, Peru, than to the local Colombian population, suggestive of cross-border spread. Nariño and Sullana are both located along the Pacific coast but also have borders with the Amazon region, which might provide an infection reservoir to both sites. Our data also shows comparable prevalence between Colombia and Peru at most of the putative resistance-conferring mutations in *pvdhfr*, *pvdhps* and *pvmdr*. However, the evidence for other *P. vivax* adaptations that differ between Colombia and Peru, highlight the risk of introducing new variants into either population that may undermine local interventions.

Our data demonstrate the potential of molecular approaches to capture new insights on local *P. vivax* transmission and adaptations within Colombia, as well as cross-border spread. However, obtaining high-quality whole genome sequencing data from *P. vivax* clinical isolates remains challenging, constraining sample size and the opportunity to investigate high-resolution spatial trends. High-throughput genotyping using approaches such as amplicon-based sequencing of SNP or microhaplotype barcodes offers a more cost-effective approach for *P. vivax* surveillance at high spatial resolution^75,76^. At high spatio-temporal density, the data generated from molecular surveillance has great potential to support the NMCP in their surveillance and response strategies to fast-track *P. vivax* elimination.

### Supplementary Information

Supplementary Information 1.

Supplementary Information 2.

Supplementary Information 3.

## Data Availability

The raw sequencing data underlying the genomes of the 64 high-quality Colombian *P. vivax* samples used in the study have all been deposited into the European Nucleotide Archive, with accession codes detailed in Supplementary Data 1. The *P. vivax* genotyping data used for the Colombian and global analyses derives from the MalariaGEN Pv4.0 dataset and is accessible as a Variant Calling Format (VCF) file (describing ~ 4.5 million variants in 1,895 worldwide *P. vivax* genomes) on the MalariaGEN website at https://www.malariagen.net/data/open-dataset-plasmodium-vivax-v4.0.
