# Supplementary material for: Genomics of Plasmodium vivax in Colombia reveals evidence of local bottle-necking and inter-country connectivity in the Americas
Source: Sci Rep. 2023 Nov 13;13:19779. doi: 10.1038/s41598-023-46076-1 (PMC10643449; doi:10.1038/s41598-023-46076-1)
Supplement: Supplementary file 1 — Supplementary Information 1. [file 41598_2023_46076_MOESM1_ESM.pdf]

Supplementary Information for:

Genomics of *P. vivax* in Colombia reveals evidence of local bottle-necking and inter-country connectivity in the Americas

Edwin Sutanto[1,2]*, Zuleima Pava[3]*, Diego F Echeverry[4,5], Tatiana M Lopera-Mesa[6], Lidia Madeline Montenegro[6], Maria F Yasnot-Acosta[7], Ernest Diez Benavente[8], Richard D Pearson[9], Sócrates Herrera[10], Myriam Arévalo-Herrera[10,11], Hidayat Trimarsanto[2,3], Angela Rumaseb[3], Rintis Noviyanti[2], Dominic P Kwiatkowski[9]**, Ric N Price[3,12,13], Sarah Auburn[3,12,13]***

1. Exeins Health Initiative, Jakarta, Indonesia
2. Eijkman Institute for Molecular Biology, Jakarta, Indonesia
3. Menzies School of Health Research and Charles Darwin University, Darwin, Australia
4. Departamento de Microbiología, Universidad del Valle, Cali, Colombia
5. International Training and Medical Research Center (CIDEIM), Cali, Colombia
6. Universidad de Antioquia, Medellín, Colombia
7. Grupo de Investigaciones Microbiológicas *y* Biomédicas de Córdoba (GIMBIC), Universidad de Córdoba, Monteria, Colombia
8. Laboratory of Experimental Cardiology, Department of Cardiology, University Medical Center Utrecht, Utrecht, the Netherlands
9. Wellcome Sanger Institute, Cambridge, UK
10. Caucaseco Scientific Research Center, Cali, Colombia
11. Universidad del Valle, Cali, Colombia

12. Mahidol-Oxford Tropical Medicine Research Unit, Mahidol University, Bangkok, Thailand
13. Centre for Tropical Medicine and Global Health, Nuffield Department of Medicine, University of Oxford, Oxford, UK

*These authors made equal contributions to the study

**Deceased April 24th, 2023

***Correspondence to Associate Professor Sarah Auburn: Sarah.Auburn@menzies.edu.au

Contents

Supplementary Figure 1 3

Supplementary Figure 2 5

Supplementary Figure 3 7

Supplementary Table 1 8

Supplementary Table 2 9

Supplementary Table 3 13

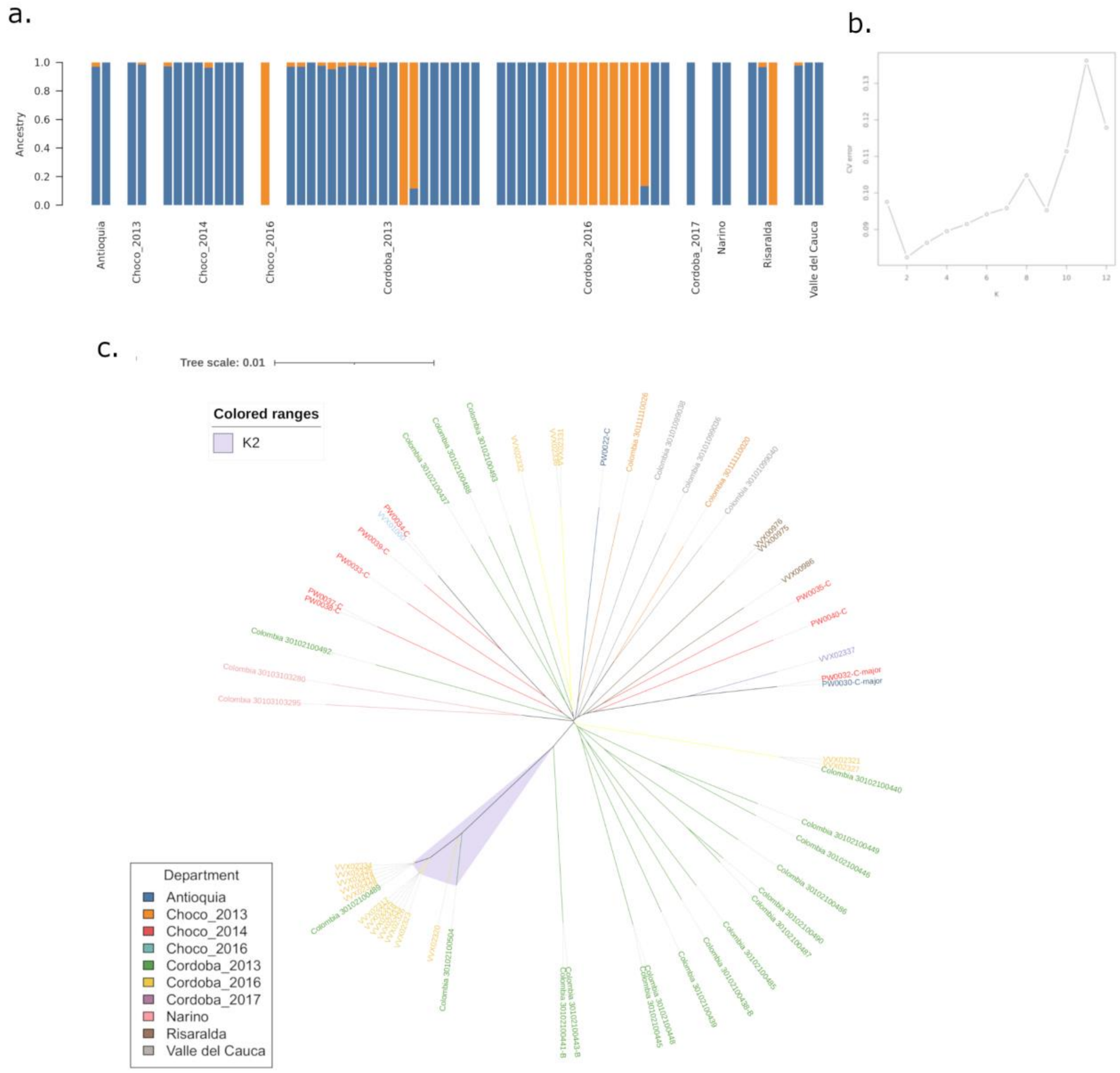

Supplementary Figure 1. Temporal patterns in infection relatedness within Colombia.

Panels a) to d) present plots similar to Figure 3, but with annotation by department and year of collection for isolates from the two major study sites: Chocó and Córdoba. Panel b) illustrates ADMIXTURE-based CV error at different values of K, revealing K=2 as the lowest CV error. Panel a) illustrates the proportionate ancestry of samples (vertical bars) to each of the two subpopulations at

K=2. At K=2, the majority of infections have predominant ancestry to K1 (in blue), with the exception of the 2016 Córdoba samples, majority of which had predominant ancestry to K2 (orange). Panel c) presents an unrooted neighbour-joining (NJ) tree illustrating the high relatedness amongst the K2 infections (mostly from 2016). All plots present monoclonal Colombian infections only.

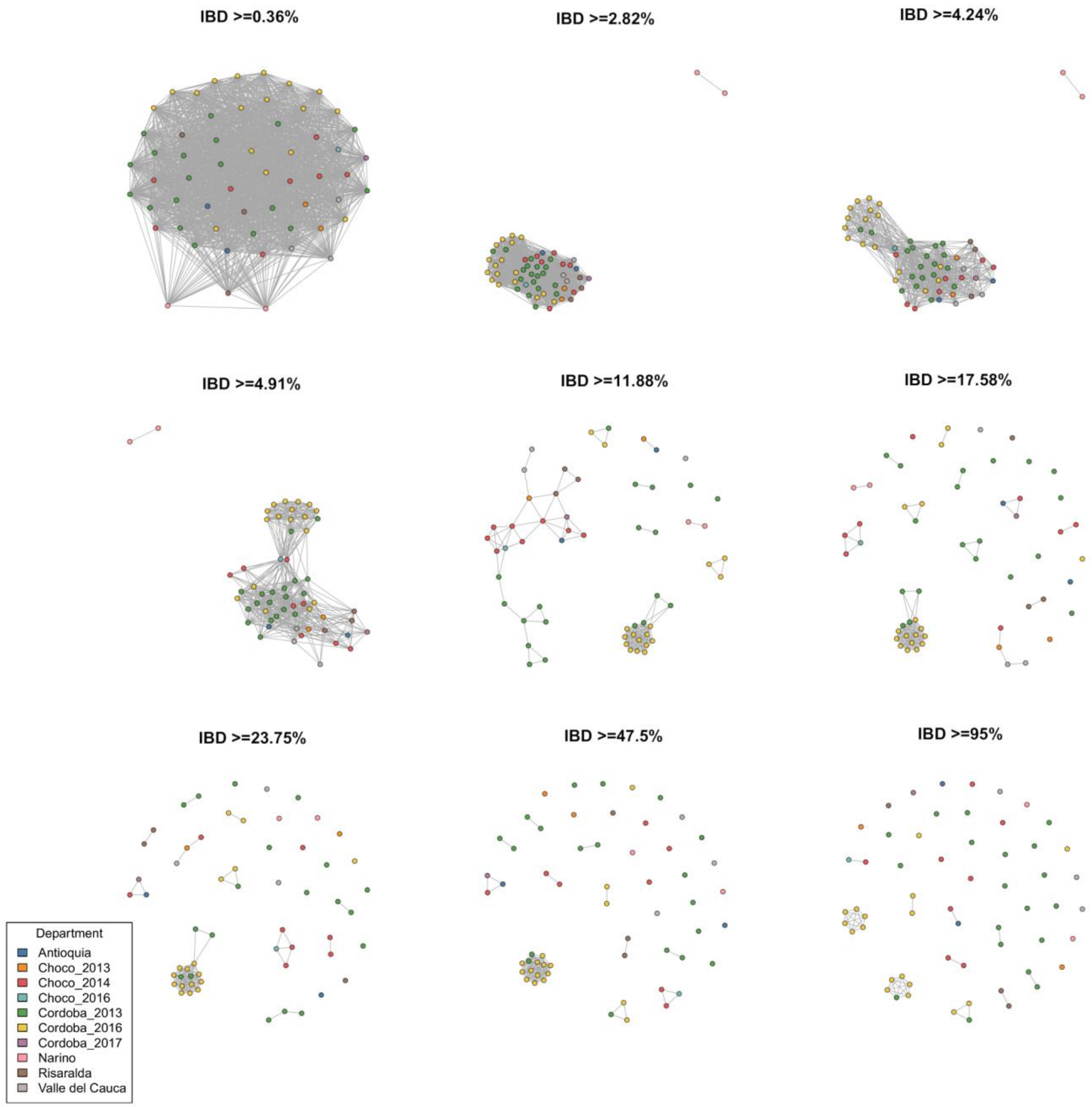

Supplementary Figure 2. Spatio-temporal patterns in infection relatedness within Colombia at increasing IBD thresholds.

The cluster networks illustrate connections between infections sharing minimum genomic IBD ranging from 0.36% (allowing capture of distant relatives) to 95% (only capturing near-identical infections). The 2016 Córdoba infections (mostly K2 infections) have the greatest connectivity. However, many

infections from a range of departments and time points exhibit relatively recent connectivity; at ~12% IBD, except for 3 samples, all infections have connectivity with at least one other infection. All plots present monoclonal Colombian infections only.

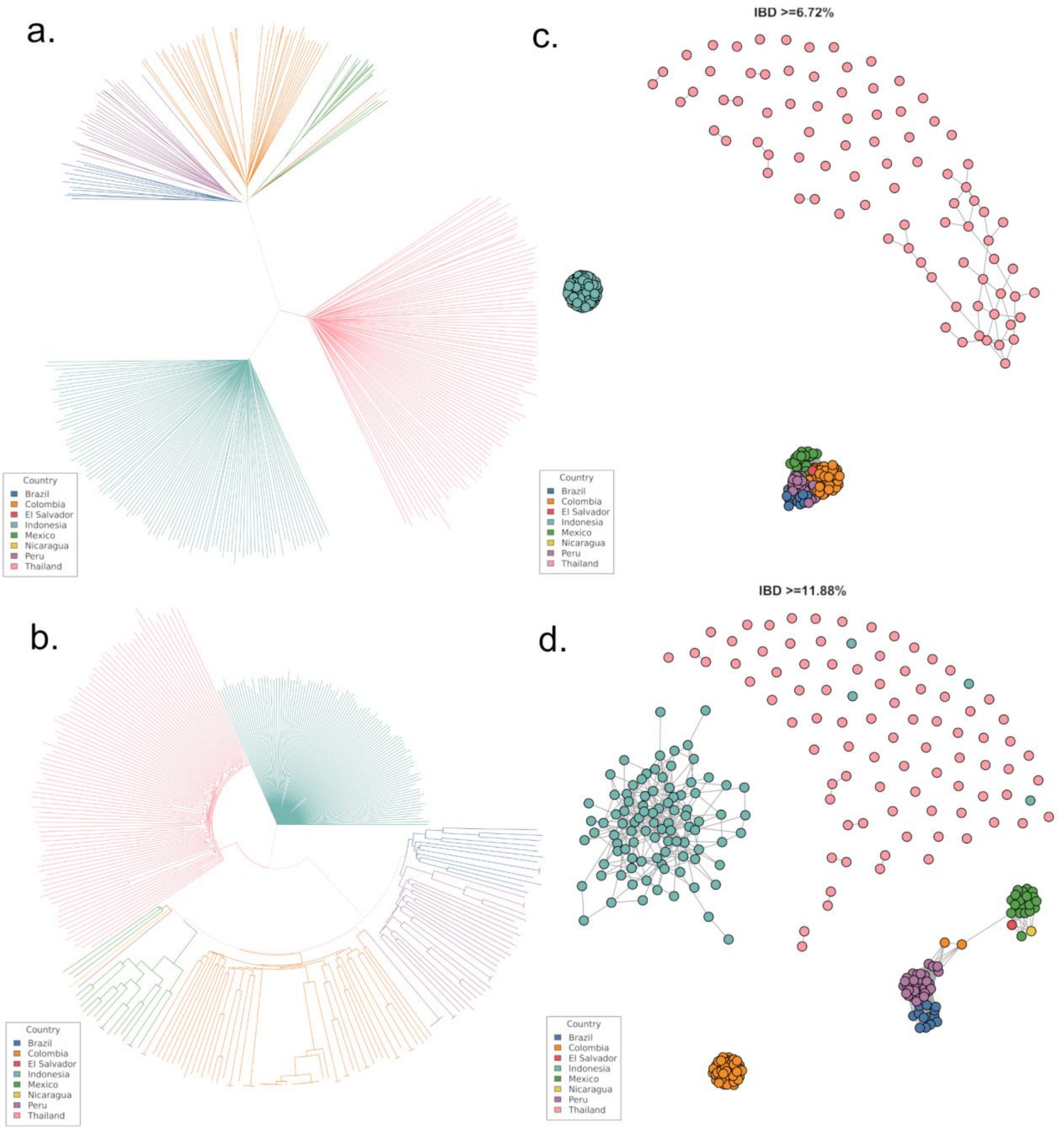

Supplementary Figure 3. Colombian infection relatedness relative to other global populations.

Panels a) and b) illustrate rooted and unrooted neighbour-joining (NJ) trees, respectively. Panels c) and d) illustrate Identity by Descent (IBD)-based cluster networks illustrating connections at minimum

thresholds of ~6 and ~12% genomic IBD, respectively. The Central and South American populations exhibit higher relatedness than the Asian populations. All plots present monoclonal infections only.

Supplementary Table 1. Summary statistics on Identity by Descent within and between countries in the Americas

Countries	Min	1st Qu.	Median	Mean	3rd Qu.	Max.
Colombia-Colombia	0.000	0.041	0.054	0.109	0.067	1.000
Colombia-Brazil	0.000	0.002	0.004	0.004	0.006	0.014
Colombia- Peru	0.000	0.002	0.004	0.005	0.006	0.097
Colombia-Mexico	0.000	0.001	0.003	0.004	0.005	0.018
Brazil-Brazil	0.035	0.059	0.077	0.107	0.092	0.999
Brazil-Peru	0.000	0.013	0.017	0.018	0.024	0.056
Brazil-Mexico	0.000	0.002	0.004	0.004	0.005	0.016
Peru-Peru	0.006	0.040	0.064	0.100	0.103	1.000
Peru-Mexico	0.000	0.002	0.004	0.004	0.006	0.012
Mexico-Mexico	0.004	0.022	0.315	0.297	0.455	1.000

Pairwise Identity by Descent (IBD) data is presented on n=122 independent single clone *P. vivax* samples from Colombia (n=58), Brazil (n=13), Peru (n=32) and Mexico (n=19). Data from El Salvador and Nicaragua was excluded owing to small sample size (n=1 in each population).

Supplementary Table 2. Summary of multi-SNP regions with evidence of extended haplotype homozygosity

Test[1]	Test pops	Signal[2]	Signal pop[3]	Chromosome	Coordinates	Length (bp)	No. SNPs	No. genes	Coordinate and function of maximum test score
Rsb	**Colombia vs Brazil**	1	Brazil	PvP01_05_v1	331740-338447	6708	33	2	331740: Intergenic
		2	Brazil	PvP01_05_v1	368473-368884	412	8	1	368533: PVP01_0508200 (HECT-like E3 ubiquitin ligase, putative)
		3	Brazil	PvP01_05_v1	410368-411133	766	12	1	410368: PVP01_0509200 (ankyrin-repeat protein, putative)
		4	Brazil	PvP01_05_v1	968076-998445	30370	72	0	996872: Intergenic
		5	Brazil	PvP01_05_v1	1033895-1046171	12277	64	3	1037784: PVP01_0525600 (conserved protein, unknown function)
		6	Brazil	PvP01_05_v1	1060465-1067336	6872	3	1	1066232: PVP01_0526300 (conserved Plasmodium protein, unknown function)
		7	Brazil	PvP01_05_v1	1093337-1109944	16608	16	1	1096046: PVP01_0526800 (conserved Plasmodium protein, unknown function)
		8	Brazil	PvP01_06_v1	286469-289897	3429	4	1	286934: PVP01_0606900 (conserved Plasmodium protein, unknown function)
		9	Colombia	PvP01_07_v1	1219896-1219915	20	3	1	1219896: PVP01_0728900 (merozoite surface protein 1)
		10	Brazil	PvP01_08_v1	529128-529356	229	5	0	529356: Intergenic
		11	Brazil	PvP01_10_v1	1095578-1118212	22635	28	4	1117625: PVP01_1025500 (guanidine nucleotide exchange factor, putative)
		12	Brazil	PvP01_10_v1	1133974-1139050	5077	10	1	1139050: Intergenic

		13	Brazil	PvP01_10_v1	1160516-1161013	498	3	1	1161013: PVP01_1026700 (subtilisin-like ookinete protein SOPT, putative)
		14	Brazil	PvP01_10_v1	1242124-1257363	15240	17	2	1256540: PVP01_1029200 (conserved Plasmodium protein, unknown function)
		15	Brazil	PvP01_10_v1	1270515-1281406	10892	26	4	1275305: Intergenic
		16	Brazil	PvP01_10_v1	1302296-1309794	7499	14	1	1307150: PVP01_1030300 (myosin B, putative)
		17	Brazil	PvP01_10_v1	1383677-1408029	24353	130	4	1388438: Intergenic
		18	Brazil	PvP01_14_v1	2862026-2862061	36	3	1	2862061: PVP01_1467200 (osmiophilic body protein G377, putative)
		19	Brazil	PvP01_14_v1	2901596-2920813	19218	122	5	2914950: Intergenic
Rsb	**Colombia vs Thailand**	1	Thailand	PvP01_02_v1	140333-154843	14511	244	2	149267: Intergenic
		2	Thailand	PvP01_05_v1	1066227-1067363	1137	4	1	1067159: PVP01_0526300 (conserved Plasmodium protein, unknown function)
		3	Thailand	PvP01_05_v1	1093275-1096057	2783	14	1	1093275: PVP01_0526800 (conserved Plasmodium protein, unknown function)
		4	Colombia	PvP01_09_v1	756189-756416	228	4	1	756374: PVP01_0916800 (pyridoxine biosynthesis protein PDX2, putative)
		5	Colombia	PvP01_11_v1	676991-677295	305	8	1	677222: PVP01_1115800 (conserved Plasmodium protein, unknown function)
		6	Colombia	PvP01_11_v1	1423431-1431629	8199	8	2	1423431: PVP01_1133300 (conserved oligomeric Golgi complex subunit 4, putative)
		7	Colombia	PvP01_11_v1	1557314-1557384	71	3	1	1557328: PVP01_1136400 (6-cysteine protein P12)

		8	Colombia	PvP01_12_v1	324253-324862	610	13	1	324601: PVP01_1208000 (6-cysteine protein P47)
		9	Colombia	PvP01_12_v1	2873923-2874598	676	3	1	2874006: PVP01_1268600 (metacaspase-2, putative)
		10	Thailand	PvP01_13_v1	808294-815132	6839	31	2	814651: PVP01_1317300 (conserved Plasmodium protein, unknown function)
Rsb	**Colombia vs Indonesia**	1	Indonesia	PvP01_10_v1	239319-326974	87656	1208	20	318227: PVP01_1007200 (conserved Plasmodium protein, unknown function, pseudogene)
		2	Indonesia	PvP01_10_v1	343467-347540	4074	7	1	345358: PVP01_1007600 (ADP-ribosylation factor GTPase-activating protein 2, putative)
		3	Colombia	PvP01_11_v1	1265741-1266097	357	5	1	1266088: PVP01_1129500 (PIMMS43 protein, putative)
		4	Indonesia	PvP01_14_v1	2861540-2928574	67035	714	14	2862053: PVP01_1467200 (osmiophilic body protein G377, putative)
Rsb	**Colombia vs Peru**	1	Peru	PvP01_05_v1	210189-212471	2283	5	0	210191: Intergenic
		2	Peru	PvP01_06_v1	838830-838838	9	4	1	838830: PVP01_0620700 (conserved Plasmodium protein, unknown function)
		3	Peru	PvP01_08_v1	1338680-1339247	568	7	0	1339247: Intergenic
		4	Peru	PvP01_09_v1	337963-339487	1525	29	1	339133: PVP01_0905700 (calcium/calmodulin-dependent protein kinase, putative)
		5	Peru	PvP01_09_v1	1320736-1322087	1352	4	1	1321747: Intergenic
		6	Peru	PvP01_11_v1	75780-75861	82	4	1	75803: PVP01_1101500 (Plasmodium exported protein, unknown function)

7	Peru	PvP01_12_v1	716454-717078	625	6	1	717004: PVP01_1217200 (conserved Plasmodium protein, unknown function)
8	Peru	PvP01_12_v1	843439-843517	79	4	1	843439: PVP01_1221100 (U3 small nucleolar RNA-associated protein 4, putative)
9	Peru	PvP01_12_v1	1420836-1429698	8863	5	0	1429698: Intergenic
10	Colombia	PvP01_12_v1	2580337-2580601	265	6	1	2580439: PVP01_1262700 (1-acyl-sn-glycerol-3-phosphate acyltransferase, putative)

[1] No multi-SNP signals were detected with the *iHS* test. [2] SNP-based p-values corresponding with thresholds of *iHS* ≥ 4 and *Rsb* ≥ 5 were considered significant, and candidate signals of selection were defined as regions with a minimum of 3 significant SNPs within a 10 kb window. Data is derived from independent single clone *P. vivax* samples with single representation of infection clusters exhibiting pairwise IBD≥0.95; Colombia (n=39), Brazil (n=12), Peru (n=26), Mexico (n=17), Thailand (n=85) and Indonesia (n=98).

Supplementary Table 3. Copy number in the Colombian samples at globally detected *P. vivax* tandem duplications

MalariaGEN sample identifier	Department	DBP[1] copy number	PvP01_09[2] copy number	MDR1[3] copy number	PVP01_1468200[4] copy number
Colombia_30101099036	Valle del Cauca	NA	NA	NA	NA
Colombia_30101099038	Valle del Cauca	NA	NA	NA	NA
Colombia_30101099040	Valle del Cauca	NA	NA	NA	NA
Colombia_30102100437	Cordoba	NA	NA	NA	NA
Colombia_30102100438-B	Cordoba	NA	NA	NA	NA
Colombia_30102100439	Cordoba	NA	NA	NA	NA
Colombia_30102100440	Cordoba	NA	NA	NA	NA
Colombia_30102100441-B	Cordoba	NA	NA	NA	NA
Colombia_30102100443-B	Cordoba	NA	NA	NA	NA
Colombia_30102100445	Cordoba	NA	NA	NA	NA
Colombia_30102100446	Cordoba	NA	NA	NA	NA
Colombia_30102100448	Cordoba	NA	NA	NA	NA
Colombia_30102100449	Cordoba	NA	NA	NA	NA
Colombia_30102100485	Cordoba	NA	NA	NA	NA
Colombia_30102100486	Cordoba	NA	NA	NA	NA
Colombia_30102100487	Cordoba	NA	NA	NA	NA
Colombia_30102100488	Cordoba	NA	NA	NA	NA
Colombia_30102100489	Cordoba	NA	NA	NA	NA
Colombia_30102100490	Cordoba	NA	NA	NA	NA
Colombia_30102100492	Cordoba	NA	NA	NA	NA
Colombia_30102100493	Cordoba	NA	NA	NA	NA
Colombia_30102100504	Cordoba	NA	NA	NA	NA
Colombia_30103103280	Narino	NA	NA	NA	NA
Colombia_30103103295	Narino	NA	NA	NA	NA
Colombia_30111110020	Choco	NA	NA	NA	NA
Colombia_30111110026	Choco	NA	NA	NA	NA
PW0022-C	Antioquia	NA	NA	NA	NA
PW0033-C	Choco	1	1	1	1
PW0034-C	Choco	1	1	1	1
PW0035-C	Choco	1	1	1	1
PW0037-C	Choco	1	1	1	1
PW0038-C	Choco	1	1	1	1
PW0039-C	Choco	1	1	1	1
PW0040-C	Choco	1	1	1	1
VVX00975	Risaralda	NA	NA	NA	NA
VVX00976	Risaralda	NA	NA	NA	NA
VVX00986	Risaralda	NA	NA	NA	NA
VVX01000	Choco	NA	NA	NA	NA
VVX02317	Cordoba	NA	NA	NA	NA

VVX02318	Cordoba	1	1	1	1
VVX02319	Cordoba	1	1	1	1
VVX02320	Cordoba	1	1	1	1
VVX02321	Cordoba	1	1	1	1
VVX02323	Cordoba	1	1	1	1
VVX02324	Cordoba	1	1	1	NA
VVX02325	Cordoba	1	1	1	1
VVX02326	Cordoba	1	1	1	1
VVX02327	Cordoba	1	1	1	1
VVX02328	Cordoba	1	1	1	1
VVX02331	Cordoba	1	1	1	1
VVX02332	Cordoba	1	1	1	NA
VVX02333	Cordoba	1	1	1	1
VVX02334	Cordoba	1	1	NA	1
VVX02335	Cordoba	1	1	1	1
VVX02336	Cordoba	1	1	1	1
VVX02337	Cordoba	NA	NA	NA	NA

Copy number was determined at 4 gene regions where large (>3 Kb) tandem duplications have been detected in *P. vivax* infections in the global MalariaGEN Pv 4.0 data set: [1] Breakpoint located at PvP01_06_v1 from 980472 to 988669, with longest breakpoint length at 8179 bp; encompasses *pvdbp*. [2] Breakpoint located at PvP01_09_v1 from 392555 to 437388, with breakpoint length at 44831 bp; encompasses eight genes on chromosome 9. [3] Breakpoint located at PvP01_10_v1 from 468190 to 506339, with breakpoint length at 38134 bp; encompasses *pvmdr1* and eight other genes on chromosome 10. [4] Breakpoint located at PvP01_14_v1 from 2894706 to 2921162, with longest breakpoint length at 26452 bp; encompasses PVP01_1468200 – a conserved Plasmodium protein with unknown function. NA (not applicable) indicates samples/sites which did not meet the required read depth thresholds for robust copy number determination. Data is derived from a starting set of n=56 independent single clone *P. vivax* samples from Colombia.
